# Apoptosis, the only cell death pathway that can be measured in human diploid dermal fibroblasts following lethal UVB irradiation

**DOI:** 10.1038/s41598-020-75873-1

**Published:** 2020-11-03

**Authors:** Anne-Sophie Gary, Patrick J. Rochette

**Affiliations:** 1 Centre de Recherche du CHU de Québec-Université Laval, Axe Médecine Régénératrice, Hôpital du Saint-Sacrement, Quebec, QC Canada; 2 Centre de Recherche en Organogénèse Expérimentale de l’Université Laval/LOEX, Université Laval, Quebec, QC Canada; 3 Département d’Ophtalmologie et ORL-Chirurgie Cervico-Faciale, Université Laval, Quebec, QC Canada

**Keywords:** Cell death, Cancer

## Abstract

Ultraviolet radiation (UVR) is a major environmental genotoxic agent. In skin, it can lead to the formation of mutagenic DNA damage. Several mechanisms are in place to prevent the conversion of these DNA damage into skin cancer-driver mutations. An important mutation prevention mechanism is the programmed cell death, which can safely dispose of the damaged cells. Apoptosis is the most studied and best characterised programmed cell death, but an increasing amount of new cell death pathways are emerging. Using different pharmacological cell death inhibitors and antioxidants, we have evaluated the implication of apoptosis, necroptosis, ferroptosis and parthanatos in UVB-induced cell death in human diploid dermal fibroblasts. Our results show that apoptosis is the only known cell death mechanism induced by UVB irradiation in fibroblasts. We also showed that lethal UVB irradiation induces a PARP-dependent drastic loss of cellular metabolic activity caused by an overused of NAD+.

## Introduction

A major environmental stress for skin is ultraviolet radiation (UVR)^1^. UVR is composed of UVC (200–280 nm), UVB (280–315 nm) and UVA (315–400 nm). UVC and short UVB (< 295 nm) are extremely toxic for the cells but they are completely blocked by the ozone layer. Long UVB (295–315 nm) and UVA are poorly filtered by the ozone layer and are known to cause DNA damage and cell death to ocular and skin cells^2–6^. UVA and UVB can penetrate skin and affect the epidermis and the dermis^7,8^. UVB wavelengths lead mainly to bi-pyrimidine DNA damage, i.e. cyclobutane pyrimidine dimers (CPD) and pyrimidine (6–4) pyrimidone photoproducts (6–4 PP)^9,10^. It can also cause oxidative damage to a lesser extent^11,12^. Several mechanisms are in place to prevent these damage from being converted into keratinocyte cancer-driver mutations^13,14^. An important mutation prevention mechanism is the nucleotide excision repair (NER) system, which removes bi-pyrimidine DNA photoproducts in human cells^15^. When the extent of DNA damage is important, programmed cell death can be triggered to safely dispose of the damaged cell.

Programmed cell death is thus important for the suppression of damaged cells and is considered as an important skin cancer prevention mechanism^16^. Among regulated cell death (RCD) pathways, apoptosis is the most studied and best characterised. Apoptosis is described as a cascade of activated caspases, leading to cell death, and triggered either by the intrinsic pathway (e.g. via DNA damage) or by the extrinsic pathway (e.g. via death receptors activation)^17^. More recently, other programmed cell death pathways, such as necroptosis, ferroptosis and parthanatos, have been described and studied (reviewed in Ref.^18^). Necroptosis results from the activation of a cascade of phosphorylation involving receptor-interacting serine/threonine-proteine kinase 3 (RIPK3) and mixed lineage kinase domain-like (MLKL) kinases^19,20^. Ferroptosis is defined by the Nomenclature Committee on Cell Death 2018 as a “form of RCD initiated by oxidative perturbations of the intracellular microenvironment that is under constitutive control by anti-glutathione peroxidase 4 (GPX4) and can be inhibited by iron chelators and lipophilic antioxidants”. Parthanatos is trigger by an hyperactivation of poly(ADP-ribose) polymerase (PARP) and can be coupled to translocation of apoptosis inducing factor (AIF) from mitochondria to nucleus^21,22^. The study of cell death is complicated by the crosstalk of cell death pathways and by the fact that death pathways depend on death signal, cell type and environment^22–24^.

Apoptosis is the only pathway known to be activated by UVB in dermal fibroblasts and in epidermal keratinocytes^25,26^. However, recent studies have shown that UVR can induce other type of programmed cell death in other cell types. Indeed, UVC can induce neutrophil extracellular traps cell death (NETosis) and apoptosis simultaneously in neutrophil from human peripheral blood, with a predominance of apoptosis at low UV dose and an increase of NETosis at higher dose^27^. PARP-1 has been found to play a role in protecting human lens epithelium against low levels of UVB light, and the authors present the possibly that PARP may trigger cell death following a toxic level of radiation^28^. Also, the protein AIF has been shown to be involved in UVB-induced caspase-independent cell death in Jurka T Cell^29^. In a previous publication, we found an increased RIPK3 transcription post-UVB in fibroblasts^30^, suggesting the activation of necroptosis by UVB. Others studies have also shown that UVB-induced ROS are also involved in UVB-induced cell death and that PARP1 is involved in DNA damage response (DDR)^31–34^. Those results imply that UVR can potentially induce non-apoptotic programmed cell death in skin cells.

In this project, we have used different pharmacological cell death inhibitors and antioxidants to evaluate UVB-induced apoptosis, necroptosis, ferroptosis and parthanatos in human diploid dermal fibroblasts. Our results show that apoptosis is the only UVB-induced cell death pathway in fibroblast. We have also shown that PARP plays a non-parthanatos but rather metabolic key role in response to UVB.

## Materials and methods

All experiments in this study were performed in accordance with the Declaration of Helsinki, and the research protocol received approval by the CHU de Québec-Université Laval (Québec) institutional ethics committees for the protection of human subjects with written informed patient consent for study participation.

### Cell culture

Normal human diploid fibroblasts (NHDF) were taken from skin biopsies (mastectomy) of 4 healthy women from 18 to 38 years old (F18, F21, F23, F38). Fibroblast were cultured in Dulbecco’s modified Eagle’s Medium (DMEM; Wisent, Canada) supplemented with 5% FBS (Sigma, Canada) and 1% penicillin/streptomycin (Wisent, Canada) at 37 °C, 5% CO_2_.

### UVB irradiation and cellular treatment

NHDF were seeded at low confluency (day 1) in 6 or 12 well-plate for western blot, flow-cytometry or NAD-NADH analysis, or in 96 well-plate for cell viability assay (MTS and CellTOX). When cells reached full confluency, they were treated with either cell death inhibitors, antioxidant, or control media at the given concentration in DMEM. After 30 min of incubation, cells were irradiated in phosphate buffered saline (PBS; Wisent, Canada) with a lethal UVB dose of 20 000 J/m^2^ in 6 well-plate or 12 well-plate and with 30 000 J/m^2^ in 96 well-plate. The UVR source has been previously published by our group^6^. Briefly, it consisted of RPR-3000 lamps (emission peak 300 nm) (Southern New England Ultraviolet Co., USA) filtered through cellulose acetate to eliminate wavelengths < 295 nm (Kodacel TA-407 clear 0.015 inches; Eastman-Kodak Co., USA) (irradiance of 19 W/m^2^). After irradiation, cells were returned in the incubator in DMEM containing or not the inhibitors or the antioxidants until they were harvested or analysed.

Inhibitor used to block necroptosis was Necrosulfonamide (NSA; R&D System, UK). Ferroptosis was inhibited using Ferrostatin-1 (Ferro-1; Millipore Sigma, USA). The potent and specific inhibitor Q-VD-OPh (Q-VD; Millipore Sigma, USA) was used to inhibit caspases. Two PARP inhibitors were used: ABT888, also known as veliparib (ABT; Santa Cruz Biotechnology, USA) and 3-Aminobenzamide (3-ABA; Millipore Sigma, Germany). α-Tocopherol (α-Toc; Sigma, Germany) and *N*-acetylcysteine (NAC; Sigma, China) were used as general antioxidant, liposoluble and hydrosoluble respectively. Ethyl Sorbate was used as a triplet quencher (EthS; Millipore Sigma, USA).

### Flow cytometry

After irradiation, NHDF were harvested 0–6 h post-UVB. Cells were then labeled with propidium iodide (PI) and Annexin V-FITC to analyse cell death by FACS, using the kit Annexin FITC/PI (Dead Cell Apoptosis Kit with Annexin V FITC and PI for flow cytometry, Invitrogen, US). Irradiated cells (UVB) were compared to un-irradiated cells (NoUV). Total cell death was measured by adding the percentage of PI + / Annexin V– cells, PI- / Annexin V + cells and double positive cells. Annexin V binds to externalized phosphatidylserine of dying cells and PI enter cells when membranes are permeabilized.

### Metabolic activity assay

MTS assay was performed (CellTiter 96 Aqueous non-radioactive Cell proliferation assay, Promega, USA) to assess metabolic activity, as describe by the manufacturer. Briefly, after UVB irradiation, cells were incubated at 37 °C for 0–24 h. Metabolic activity was then assessed by adding MTS reagents directly on cells. After 30 min to 1 h of incubation with the MTS reagent, absorbance at 490 nm was measured. For each time point, irradiated cells (UVB) were normalised on un-irradiated cells (NoUV) of the same condition. Cells treated with the different inhibitors were compared to cells treated with the vehicle Dimethyl Sulfoxide (DMSO; Sigma, UK).

### Western Blot analysis

After treatment and irradiation of NHDF, cells were harvested 0–12 h post-UVB. Cytosolic or total proteins were then quantified using BCA quantification (Pierce BCA Protein Assay Kit, ThermoFisher Scientific, USA). Proteins were run on SDS-PAGE and visualised by SuperSignal West Pico PLUS Chemiluminescent Substrate (Thermo Fisher Scientific, USA). Membrane were scanned using the C-DiGit Blot Scanner (LI-COR Biosciences, USA) and analysed with Image Studio Lite software (LI-COR Biosciences, USA).

PAR and PARP analysis: To analyse PAR chain and PARP polymerase, cells were first wash in PBS and then harvest on ice directedly in RIPA buffer (1% NP 40, 0.5% Sodium Deoxycholate, 0.1% SDS, 150 mM NaCl, 50 mM Tris HCl pH8) complemented with cOmplete, EDTA-free Protease Inhibitor Cocktail (Roche, Germany) and phosphatase inhibitor PhosSTOP (Roche, Germany) using a cell scrapper. α-PAR (10H, 100ug/mL, 1:500, Santa Cruz Biotechnology Cat# sc-56198, RRID: AB_785249) and α-PARP (F2, 200ug/mL, 1:500, Santa Cruz Biotechnology Cat# sc-8007, RRID:AB_628105) were used in Western blot.

AIF analysis: To analyse AIF, cell pellets were harvested, and proteins were extracted from cytosol, mitochondria, and nucleus, using the Cell fractionation kit—Standard (Abcam, UK), according to the manufacturer. Nuclear proteins were resuspended in RIPA buffer. α-AIF (E-1, 200 µg/mL, 1:1000, Santa Cruz Biotechnology Cat# sc-13116, RRID:AB_626654) was used in Western blot. AIF was also used as a mitochondrial control by simultaneously scanning the membranes from the 3 fractions. In the same way, α-Tubulin (DM1A, 1:500, Abcam Cat# ab7291, RRID:AB_2241126) and α-Lamin A/C (4C11, 1:500, Cell Signalling Technology Cat# 4777, RRID:AB_10545756) were used as cytosolic and nuclear controls, respectively.

### NAD+ and NADH quantification

Nicotinamide adenine dinucleotide (NAD)/NADH assay kit (Abcam, UK) was used as described by the manufacturer. Briefly, following UVB irradiation, cells were washed twice with PBS and 3 × 10^6^ cells were harvested directly in 350 µl of Extraction Buffer provided by the kit. After two thaw/freeze cycles, samples were centrifugated and supernatants containing NAD-NADH were collected. A protein quantification was performed by BCA quantification (Pierce BCA Protein Assay Kit, ThermoFisher Scientific, USA). Same amount of proteins was used for each condition. Samples were divided, half was used to heat degrade NAD+, and the other half represents total NAD. The samples for total NAD was diluted in Extraction Buffer. Samples were then mixed with reaction buffer and Cycling Enzyme to transform all NAD+ in NADH, allowing the measure of total NAD (NADt). Concentration of NAD+ and NADH was then calculated by comparison to NADH standard curve.

### Cell death assay (CellTOX)

Following UVB exposure, a cell death assay was performed (CellTox Green Cytotoxicity Assay; Promega, USA) according to the manufacturer protocol. Briefly, media supplemented with the different cell death inhibitors was added after UVB exposure, the fluorescent dye measuring changes in membrane integrity occurring as a result of cell death was added directly in media. Fluorescence (excitation peak at 480 nm, emission peak at 530 nm) was then measured at different time-point post-UVB (0 h, 6 h, 12 h and 24 h). Value from un-irradiated cells (NoUV) were subtracted to fluorescence from irradiated cells to remove the background. Fluorescence of each condition was relative to the 0 h post irradiation.

### Statistical analysis

Data are presented with mean ± SD (or ± SEM if specified), statistical significance was assessed using repeated measures (mixed model) two-way ANOVA with a Bonferroni post-test to compare the indicated experimental groups at different time points. The “N” indicates the number of human primary cultures used in each experiment. At least duplicates for each culture were averaged in each experiment to generate one value per culture. Then values from “N” culture were average and SD was calculated to generate data point within each experiment. A p-value < 0.05 was considered statistically significant. GraphPad Prism8 software (GraphPad Software Inc., San Diego, CA, USA) was used to determine statistical significance.

## Results

### UVB-induced early cell death

**Figure 1:**
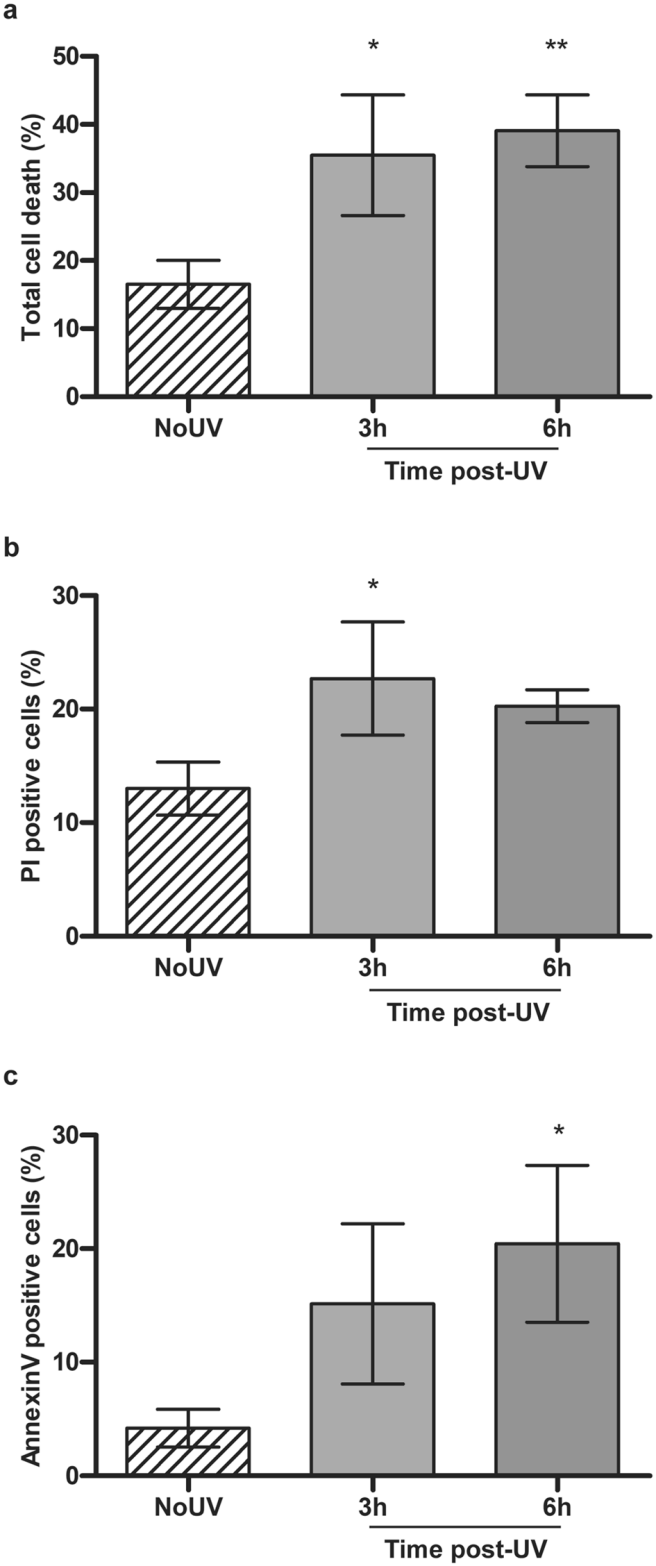
UVB-induced early cell death in primary human fibroblasts. Using Annexin V-FITC and PI labelling, we assessed cell death in fibroblasts at 3 h and 6 h following an irradiation with 20 kJ/m^2^ UVB. The fluorescence signal in each cell was assessed by FACS. (**a**) Total cell death is the addition of Annexin V+/PI− cells, Annexin V+/PI+ cells and Annexin V−/PI+ cells. (**b**) PI positive cells is composed of PI+/Annexin V+ or − cells. (**c**) Annexin V positive cells are Annexin V+ and PI−. NoUV are unirradiated control cells. UVB irradiation of fibroblasts leads to cell death as early as 3 h. Percentage is representative of a population of at least 8000 cells. N = 3, *p-value < 0.05.

The apoptotic pathway can be activated by intra or extra-cellular stresses. UVB radiation can induce apoptosis in keratinocytes and fibroblasts^35–37^. In fact, UVB radiation triggers the intrinsic and extrinsic pathway by damaging DNA, inducing oxidative stress and activating cell death receptors, causing a cascade of events leading to caspases activation^25^. Apoptosis can be visualised using Annexin V and propidium iodide (PI). We used this method to visualize early cell death in NHDF, 3 h and 6 h after UVB exposure. UVB irradiation increased PI positives cells from 13 to 22.7% and Annexin V positives cells from 4.2 to 16.2% at 3 h and 6 h post irradiation, respectively. The total cell death (i.e. PI+/Annexin V− , PI+/Annexin V+ and PI−/Annexin V+ cells) also increased by 19% and 22.5% at 3 and 6 h post irradiation compared to unirradiated cells, respectively (Fig. 1).

### UVB-induced caspase and PARP-dependant loss of metabolic activity

**Figure 2:**
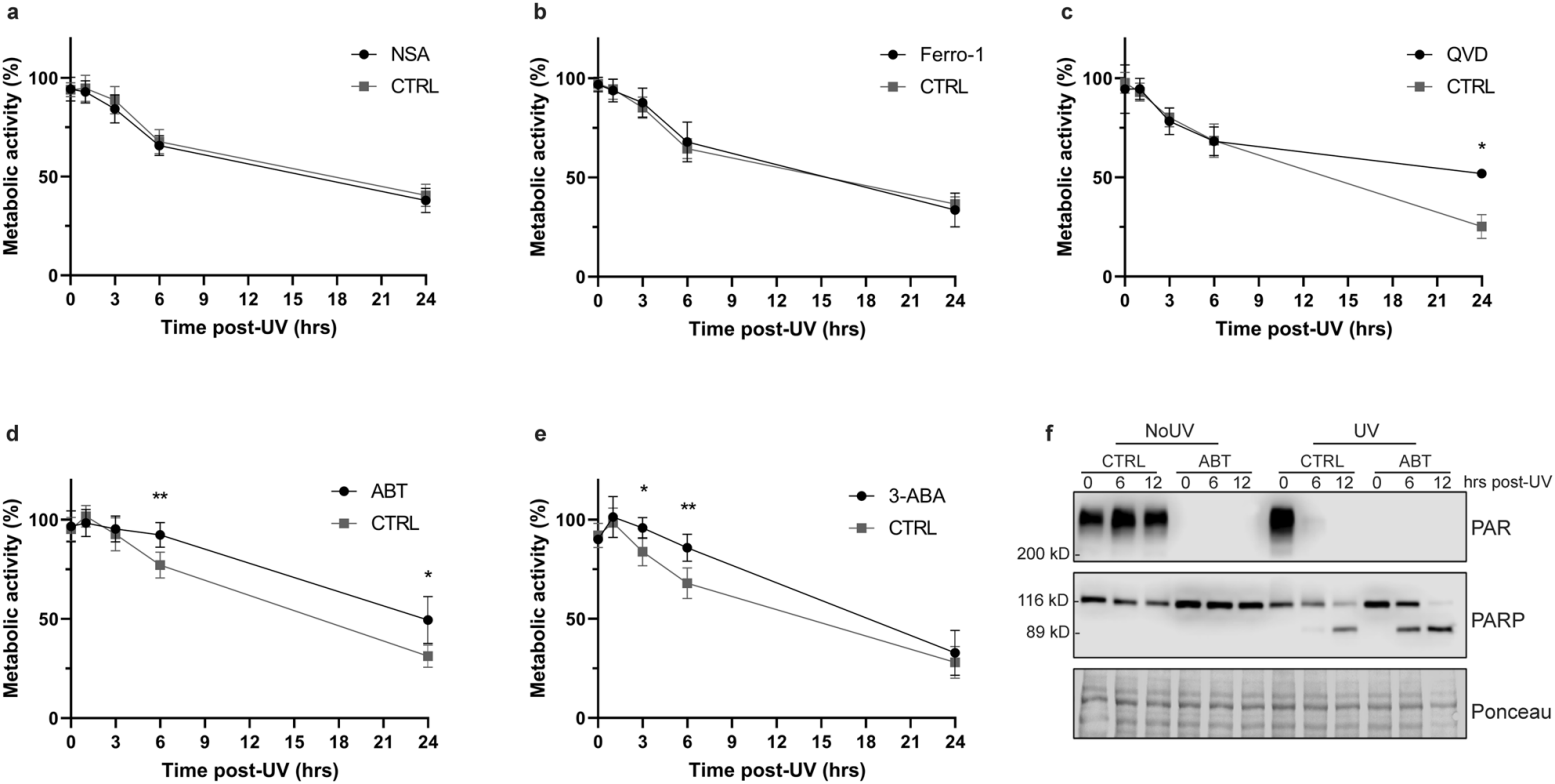
Cellular metabolic activity of UVB-irradiated primary human fibroblasts treated with different cell death inhibitors. Prior to UVB irradiation, fibroblasts were incubated 30 min with different cell death inhibitors. Cells were then irradiated in PBS using a lethal UVB dose (30 kJ/m^2^). The different cell death inhibitors used were: (**a**) The necroptosis inhibitor Necrosulfonamide (NSA, 2 μM), (**b**) the ferroptosis inhibitor Ferrostatin-1 (Ferro-1, 5 μM), (**c**) the broad caspase inhibitor Q-VD-OPh (QVD, 20 μM), (**d**) the PARP inhibitor ABT888 (ABT, 20 μM) or (**e**) the PARP inhibitor 3-Aminobenzamide (3-ABA, 0.5 mM). Cellular metabolic activity was assessed at different time points post-UVB irradiation (0, 1, 3, 6, 24 h) using MTS assay. Irradiated cells were normalised on unirradiated cells of the same condition. NSA and Ferro-1 had virtually no effect on cellular metabolism. However, QVD significantly prevent UVB-induced cellular metabolic activity loss at 24 h, ABT at 6 and 24 h and 3-ABA at 3 and 6 h. N = 4. *p-value < 0.05, **p-value < 0.01, ***p-value < 0.001. (**f**) Efficiency of ABT888 was confirmed by Western Blot, showing an inhibition of PAR formation without abolition of apoptotic PARP cleavage.

To determine the specific pathway involved in UVB-induced cell death, we used different cell death inhibitors to block necroptosis, ferroptosis, PARP-dependent cell death or apoptosis. We assessed cell viability by measuring cellular metabolic activity using a MTS assay^38^. We showed a UVB-induced decrease in metabolic activity in primary human fibroblasts as early as 3–6 h post exposition (Fig. 2). Necrosulfonamide (NSA), the inhibitor of necroptosis, and Ferrostatin-1 (Ferro-1), the inhibitor of ferroptosis, did not induce any change in metabolic activity (Fig. 2A,B). PARP inhibitors, i.e. ABT888 and 3-ABA, both prevented a loss of metabolic activity at 3–6 h post-UVB in NHDF (Fig. 2D,E). Moreover, the caspases inhibitor Q-VD-OPh prevented 26.8% of the metabolic activity loss observed at 24 h post-UVB in NHDF (Fig. 2C). Those results indicate a key role of PARP and caspases in UVB-induced metabolic activity loss in NHDF. Since caspases are specifically activated in the apoptosis cascade, the loss of metabolic activity at 24 h can be attributed to UVB-induced apoptosis. PARP is known to play an important role in DNA damage response, notably in the repair of specific UVB-induced DNA damage^31,32,39^. Our results suggest that PARP have a new role in preventing cellular metabolic activity loss early (3–6 h) after lethal UVB irradiation in NHDF. Noteworthy, ABT888 blocked PAR formation without preventing caspase-dependent cleavage of PARP1 (Fig. 2F). The prevention of metabolic activity loss using PARP inhibitors and caspase inhibitor did not occur at the same time period (i.e. early, 3–6 h and late, 24 h). This might reflect two independents events, but they can also be related. Using combination of different cell death inhibitors, we have thus focused on understanding the possible interplay between cell death pathways.

### PARP inhibition and caspase inhibition independently prevent metabolic activity loss

**Figure 3:**
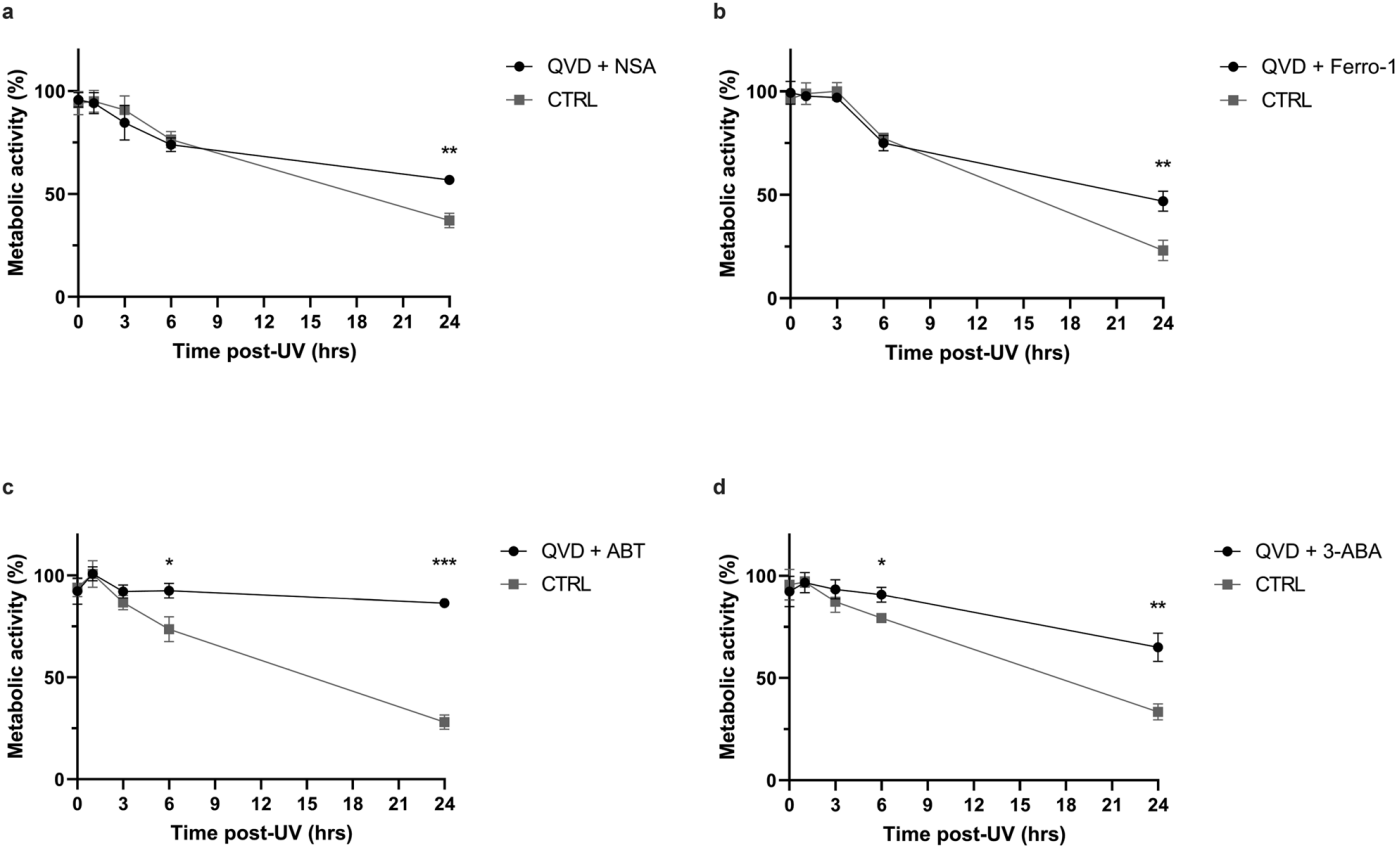
UVB induces two independent reduction of cellular metabolic activity in primary human fibroblasts. Prior to UVB irradiation, fibroblasts were incubated 30 min with different combinations of cell death inhibitors. Cells were then irradiated in PBS using a lethal UVB dose (30 kJ/m^2^). The combinations used were: (**a**) Necroptosis inhibitor Necrosulfonamide (NSA, 2 μM) and caspase inhibitor Q-VD-OPh (QVD, 20 μM), (**b**) ferroptosis inhibitor Ferrostatin-1 (Ferro-1, 5 μM) and caspase inhibitor Q-VD-OPh (QVD, 20 μM), (**c**) PARP inhibitor ABT888 (ABT, 20 μM) and caspase inhibitor Q-VD-OPh (QVD, 20 μM), (**d**) PARP inhibitor 3-Aminobenzamide (3-ABA, 0.5 mM) and caspase inhibitor Q-VD-OPh (QVD, 20 μM). Cellular metabolic activity was assessed at different time points post UVB irradiation (0, 1, 3, 6, 24 h) using MTS assay. Irradiated cells were normalised on unirradiated cells of the same condition. NSA and Ferro-1 had no effect on cellular metabolic activity loss prevention caused by QVD. However, an additive effect of QVD and PARP inhibitors (ABT and 3-ABA) on prevention of cellular metabolic activity loss caused by UVB could be described. N = 4. *p-value < 0.05, **p-value < 0.01, ***p-value < 0.001.

Programmed cell death pathways depend on cell type, environment and cell death signal. Crosstalk can occur between different cell death pathways and/or survival and cell death signalling^40,41^. We thus combined the inhibitors of necroptosis, ferroptosis and PARP with the caspases inhibitor in order to see potential compensation mechanisms or crosstalk between the different cell death pathways. Indeed, blocking apoptosis could lead to the induction of other cell death pathways to compensate. Q-VD alone had a similar effect on metabolic activity decrease caused by UVB irradiation than Q-VD combined with necroptosis inhibitor (NSA) or ferroptosis inhibitor (Ferro-1) (Fig. 3A,B). This suggests that there is no compensation mechanism by necroptosis or ferroptosis after blocking apoptosis. However, combining PARP inhibitor (ABT888) and apoptosis inhibitor (Q-VD-OPh) restored both the early and late metabolic activity loss caused by UVB irradiation in NHDF. Indeed, the effect of the two inhibitors added up, restoring the metabolic activity from 73.6 to 92.5% at 6 h post-UVB and from 28 to 86% at 24 h (Fig. 3C). We used another PARP inhibitor, 3-ABA, to confirm this result. The combination of 3-ABA and Q-VD-OPh also prevent metabolic activity loss at 6 and 24 h post irradiation, but to a lesser extent than ABT888 (Fig. 3D). Those results indicate that lethal UVB irradiation induces two independent and biphasic phase loss of metabolic activity, i.e. an early caspase-independent and PARP-dependent phase, and a late phase caspase-dependent apoptotic cell death. This effect is UVB dose independent (Supplementary Fig. S1).

### UVB-induced cell death is independent of oxidation

**Figure 4:**
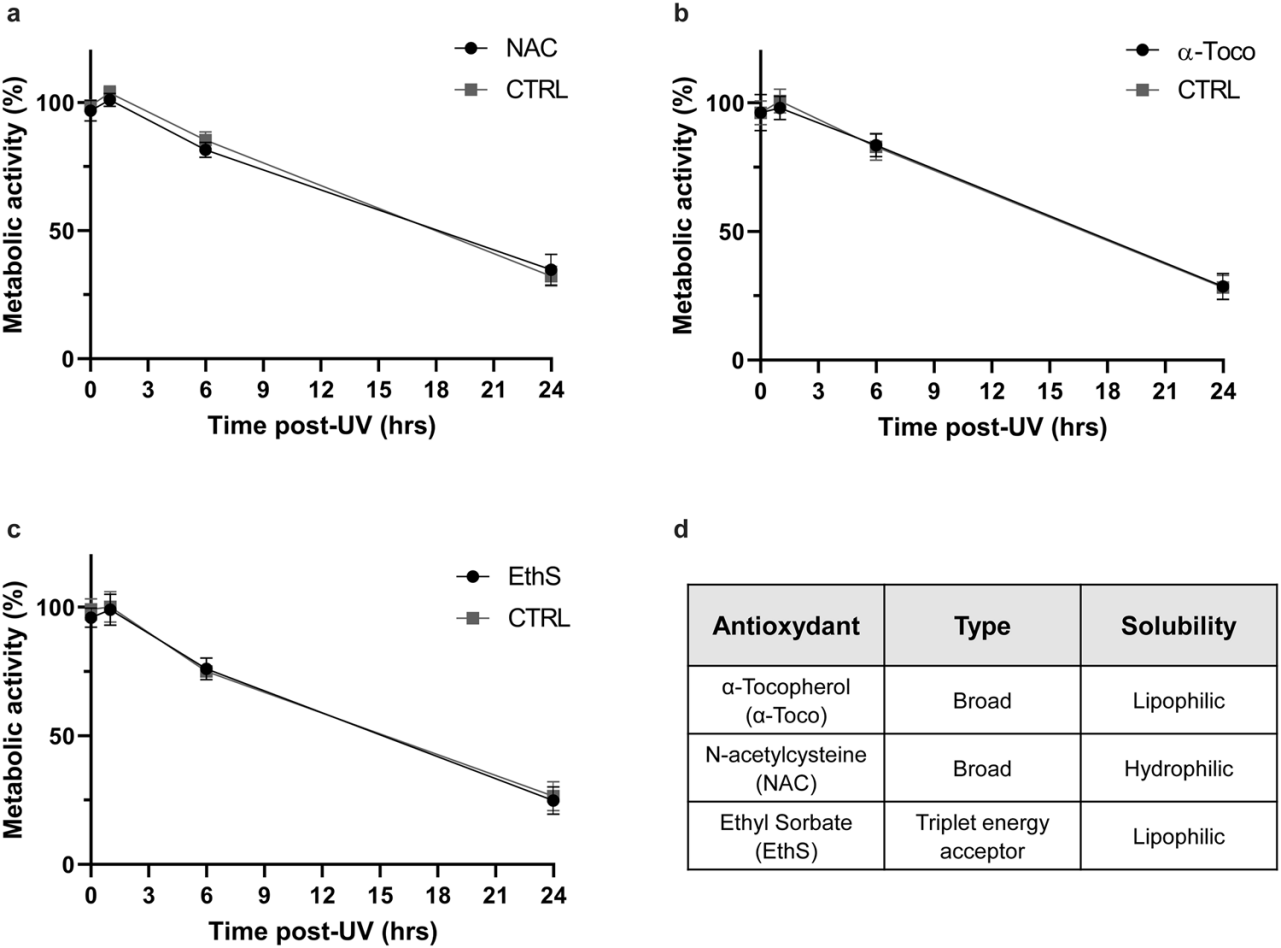
UVB-induced cell death is independent of oxidation in primary human fibroblasts. Prior to UVB irradiation, fibroblasts were incubated 30 min with different antioxidants. Cells were then irradiated in PBS using a lethal UVB dose (30 kJ/m^2^). The different antioxidant used were: (**a**) Broad-spectrum oxidation inhibitor *N*-acetylcysteine (NAC, 2.5 mM), (**b**) broad-spectrum oxidation inhibitor α-Tocopherol (α-Toco, 10 μM), (**c**) triplet energy acceptor Ethyl Sorbate (EthS, 5 μg/μl). Cellular metabolic activity was assessed at different time points post UVB exposition (0, 1, 6, 24 h) using a MTS assay. Irradiated cells were normalised on unirradiated cells of the same condition. None of the antioxidant tested had an effect on UVB-induced cellular metabolic activity loss. N = 4. (**d**) Table of antioxidants used, their type and solubility.

Signal for UVB-induced cell death can come from direct DNA damage, oxidative stress or the activation of cell death receptors (review in Refs.^25,35^). We assessed the implication of oxidation in metabolic activity loss observed post UVB irradiation to determine the signal for early and late UVB-induced cell death. We used two broad-spectrum antioxidants, i.e. *N*-acetylcysteine (NAC) and α-Tocopherol, and a triplet acceptor, Ethyl Sorbate (Fig. 4D). Neither antioxidants nor triplet quencher had an effect on early or late UVB-induced loss of metabolic activity (Fig. 4A–C). This strongly suggests that oxidation does not play a significant role in UVB-induced apoptosis or PARP-dependant loss of metabolic activity in NHDF.

### Total NAD is depleted by the UVB-induced PARP activation

**Figure 5:**
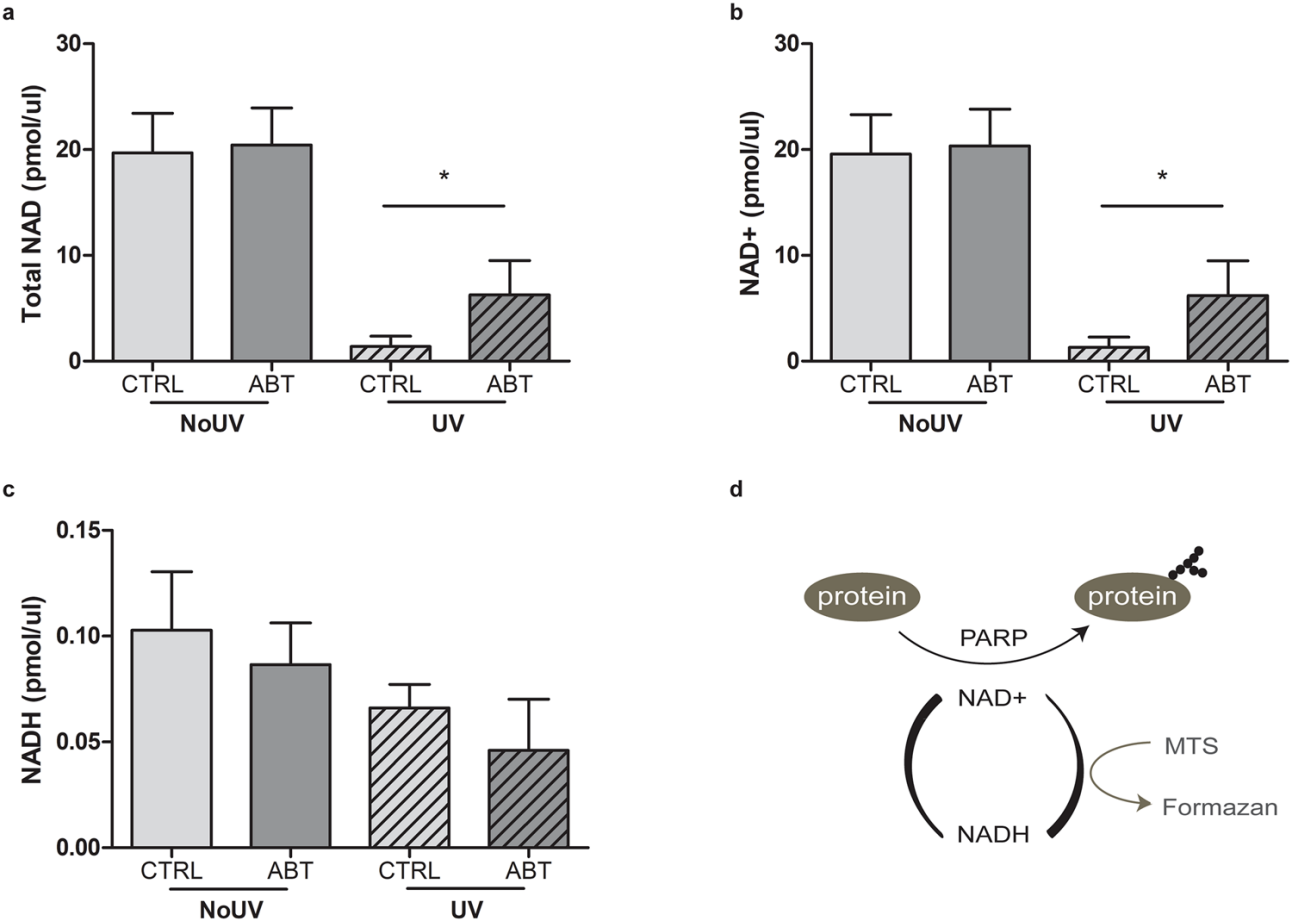
The UVB-induced total NAD and NAD+ pool decrease can partially be restored using PARP inhibitor. Prior to UVB irradiation, fibroblasts were incubated 30 min with or without ABT888 (ABT, 20 μM). Cells were then UVB irradiated with 20 kJ/m^2^ (UV) or not (NoUV) in PBS. 6 h post exposition, cells were harvested to quantify NAD/NADH. Same amount of proteins was taken to compare the different conditions, i.e. NoUV CTRL, NoUV ABT, UV CTRL and UV ABT. (**a**) Concentration of Total NAD and (**b**) concentration of NADH were measured. Concentration of NAD+ was deduced by subtracting NADH to Total NAD in (**c**). We found that UVB irradiation significantly reduces total NAD and NAD+, but the use of PARP inhibitor ABT significantly prevent this loss. N = 4, *p-value < 0.05. (**d**) Schematic representation of the link between NAD+ consumption, PARP1 and the MTS assay.

It has been previously shown that the PARP inhibitor ABT888 supresses the loss of metabolic activity induced by the depletion of NAD+ pool in HaCaT keratinocyte cells^42^. We hypothesised that UVB irradiation of NHDF leads to an over-activation of PARP, leading to a depletion of NAD+/NADH pool. Indeed, PARP uses NAD+ to PARylate targeted proteins such as the ones involved in DNA damage repair^31–33,43^. We measured the concentration of NAD+ and NADH in NHDF UVB-irradiated or not, supplemented with ABT888 or not. Total NAD drastically decreases when cells are exposed to a lethal UVB dose (Fig. 5A). Total NAD is mainly composed of NAD+ pool since the measured NADH concentration were around 200 times lower than NAD+ (Fig. 5B,C). Interestingly, ABT888 was able to restore almost a third of the lost NAD pool (Fig. 5A,C). This result suggests that a lethal UVB irradiation induces an overactivation of PARP, leading to a depletion of NAD+. This depletion in NAD+ is translated in a reduction of metabolic activity (Fig. 5D). ABT888 partly restores the pool of total NAD and NAD+ after a genotoxic stress. This also implies that the loss of metabolic activity caused by UVB irradiation, as measured by the MTS assay, could be due to NAD+ depletion and not cell death (Fig. 5D). However, the question remains as whether PARP activation and NAD reduction after UVB irradiation leads to cell death or not.

### PARP does not induce parthanatos post-UVB

**Figure 6:**
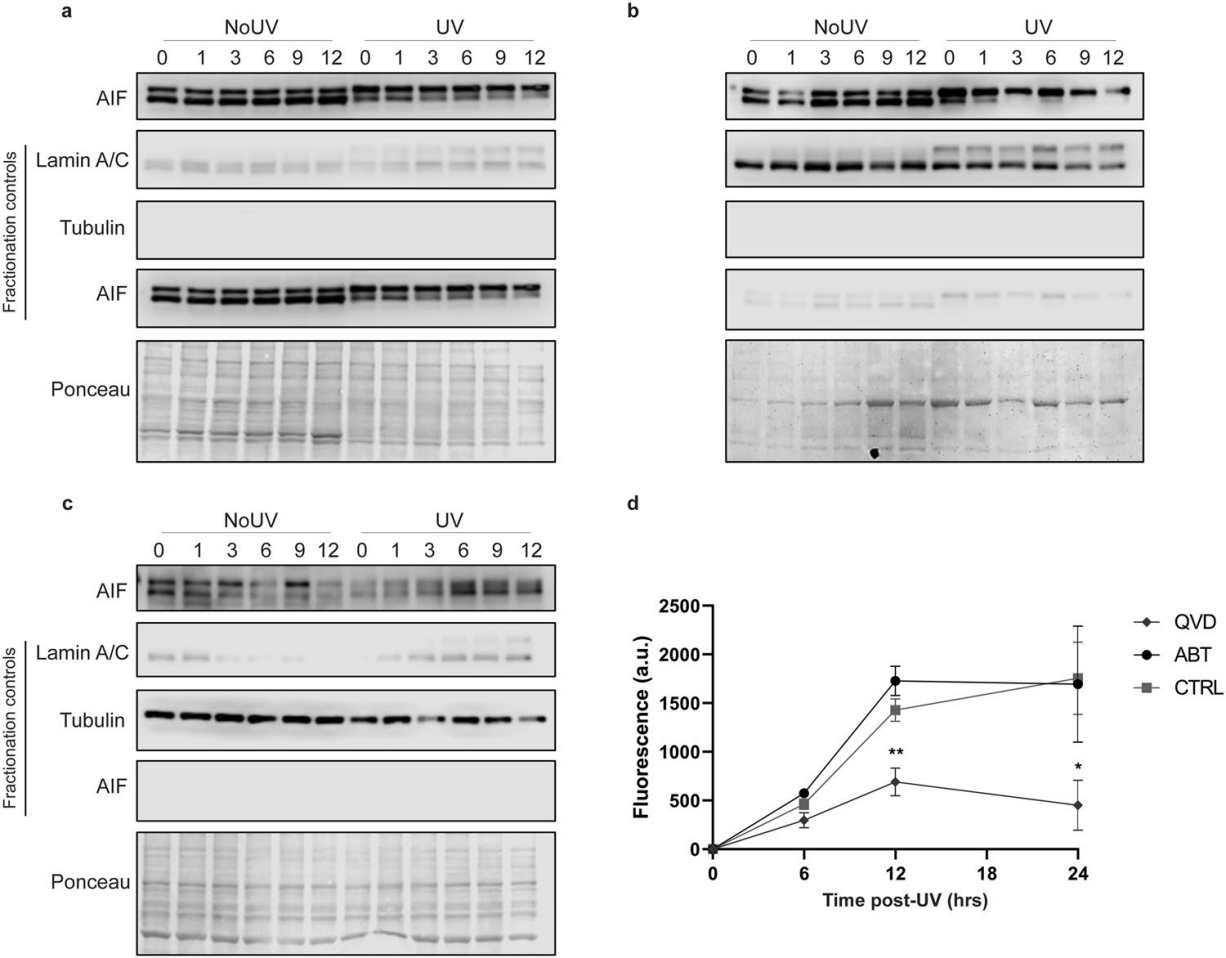
Parthanatos is not induced by UVB in primary human fibroblasts. Fibroblasts were UVB irradiated with 20 kJ/m^2^ (UV) or not (NoUV). At different time points after exposition, cells were harvested (0, 1, 3, 6, 9, 12 h) and proteins from mitochondria, nucleus and cytoplasm were separated. Western Blot against AIF using protein fractions of (**a**) mitochondria, (**b**) nucleus and (**c**) cytoplasm showed no translocation of the protein from mitochondria to nucleus. Controls of fractionation were measured by simultaneously scanning the membranes from the 3 fractions. Nuclear, cytosolic and mitochondrial controls are Lamin A/C, Tubulin and AIF, respectively. Ponceau staining has been used as loading control. (**d**) Prior to UVB irradiation at 30 kJ/m^2^, cells were incubated 30 min with ABT888 (ABT, 20 μM), Q-VD-OPh (QVD, 20 μM) or control media (CTRL). Immediately after UVB exposure cells were incubated with corresponding inhibitor and CellTOX dye. CellTOX fluorescence, proportional to cell death, was measure at different time points (0, 6, 12 and 24 h). Fluorescence from unirradiated cells (background) was subtracted to irradiated cells for the same condition. The apoptosis inhibitor QVD significantly prevents UVB-induced cell death at 12 and 24 h. However, the PARP inhibitor ABT had no effect on UVB-induced cell death. SEM, N = 4, *p-value < 0.05.

The loss of metabolic activity generally correlates with cell death^38^. PARP inhibitors help maintaining cellular metabolic activity post UVB-exposure (Fig. 2D,E). Since this mechanism is independent of caspases (Fig. 2F), UVB could induced a PARP-dependant cell death called parthanatos. Parthanatos is a cell death triggered by PARP, independent of caspases activations and dependant of AIF translocation from mitochondria to the nucleus after overactivation of PARP1 (review in Refs.^44,45^). AIF was shown to be release after parylation in the mitochondria and translocate to the cytoplasm and/or the nucleus^46^. We first investigated the translocation of AIF in NHDF after a lethal UVB dose. We observed a molecular weight shift after UVB in each fraction but could not find a translocation of AIF from the mitochondria to the cytoplasm or the nucleus (Fig. 6A–C). This molecular weight shift could be the results of a posttranslational modification post UVB but this would need further investigation, which we feel is not the purpose of this study. Since no AIF translocation was detected, we verified the role of PARP in UVB-induced cell death with a direct measure of cell death using CellTOX kit (CellTox Green Cytotoxicity Assay; Promega)^47^. The inhibition of caspases using Q-VD-OPh drastically prevent UVB-induced cell death, which confirms the involvement of apoptosis in response to lethal UVB irradiation in NHDF. However, ABT888, the PARP inhibitor, was not able to prevent UVB-induced cell death (Fig. 6D). This indicates that the PARP-dependent caspase-independent loss of metabolic activity is not related to cell death.

Our results clearly show than the only known cell death mechanism induced by lethal UVB irradiation in NHDF is apoptosis and that no compensation mechanisms backup apoptosis if this one is deficient. We found no evidence of ferroptosis, necroptosis or parthanatos in UVB-induced cell death in NHDF. We also showed that 3–6 h following lethal UVB irradiation, there is a loss of metabolic activity triggered by an overused of NAD+ due to PARP activation.

## Discussion

### Metabolic activity does not always correlate with cell death

In this study, we described 2 independent cellular metabolic activity loss in response to UVB irradiation in NHDF. The later loss of metabolic activity (24 h post-UVB) corresponds to UVB-induced caspase-dependent apoptosis (Figs. 2, 6). The earlier loss of metabolic activity (3–6 h post-UVB) is dependent of PARP activity (Fig. 2). PARP-dependent cell death (parthanatos) was thus assessed using PARP inhibitor ABT888 and this could neither prevent, nor delay UVB-induced cell death (Fig. 6). AIF, an important factor in parthanatos, was observed mainly in mitochondria, but also in cytoplasm (Fig. 6). A shift in AIF size was observed in UVB irradiated cells, suggesting that AIF have a function in response to UVB exposition but not related to parthanatos. Altogether, our results show that, even if PARP does not contribute to UVB-induced cell death, the MTS assay and NAD/NADH measure show that PARP plays an important role in metabolic activity change caused by UVB irradiation. In summary, we have clearly shown that metabolic activity does not always reflect cell death. Thus, metabolic activity using substrate reduction in formazan, such as MTT or MTS assays, should be taken with caution, especially when using PARP inhibitors.

### Apoptosis is the sole UVB-induced cell death mechanisms in NHDF

Our results showing the absence of necroptosis, ferroptosis or parthanatos strongly suggest that apoptosis is the sole UVB-induced cell death mechanism involved in NHDF (Fig. 2). Moreover, no cell death-related compensation mechanism could be found when apoptosis was inhibited (Fig. 3). Other cell death pathways exist but we excluded the study of regulated cell death (RCD) connected to immunogenicity since this component is absent in our model (ex. NETotic cell death, pyroptosis and Immunogenic cell death). Antioxidants had no effect on UVB-induced metabolic activity change (Fig. 4). Thus, RCD triggered by ROS formation such as ferroptosis, Lysosome-dependent cell death and MPT-driven necrosis can be excluded (cell death pathways review in Ref.^21^).

Previous work from our laboratory, and others, have shown that chronic UVB irradiation leads to improvement of DNA repair response in skin but results in the accumulation of unrepairable residual DNA damage^30,48,49^. We have shown that chronic low UVB dose (CLUV) treatment of NHDF induce RIPK3 transcription level, a key factor in necroptosis^30^. However, using a necroptosis inhibitor, we could not find any implication of necroptosis in UVB-induced cell death. RIPK3 could also play a role in another aspect of stress-response, such as survival signal, cytokine expression^50^, matrix production^51^ or cell cycle and cell division^52^, this should be further investigated. Chronic exposure better reflects the exposure to which we are exposed and it would be relevant to study the impact of chronic irradiation on cell death. Indeed, chronic irradiation could lead to an adaptive response or an increased sensitivity. Cells could be more sensible to apoptosis as they accumulate DNA damage or other cell death pathways could be activated in cells pre-treated with chronic irradiation.

### UVB exposure leads to PARP-dependent changes in metabolic activity

Exposure of fibroblasts to UVB irradiation induces a drastic depletion of their NAD pool, which is reverted in part by the PARP inhibitor ABT888 (Fig. 5). PARP has been shown to be hyperactivated post-UVB and use directly NAD+ to add poly ADP-ribosylation (PARylate) proteins^34^. Notably, PARP1 have a key function in DNA damage repair^31–33,39^, which could explain the observed metabolic changes following UVB. The question remains whether NAD pool depletion could accelerate cell death post-UVB. We have shown that the PARP inhibitor ABT888 is able to prevent one third of the NAD+ pool depletion, which explains the restoration of metabolic activity (Fig. 5). However, the un-prevented depleted NAD+ could play a role in cell death. NAD+ depletion could accelerate or promote cell death, which would help explaining the early cell death induced by UVB radiation observed by FACS and CellTOX assay (Figs. 1, 6). Recently, a publication highlighted mitochondrial changes mediated by UV in HaCaT cells, as well as the implication of PARP in those changes^42^, which supports our findings. Further works is necessary to decipher the precise role of mitochondria in response to genotoxic stress such as the one induced by UVB.

To conclude, we have shown that elimination of damaged cells following lethal dose of UVB relies only on apoptosis. UVB-induced cell death is an important anti-tumoral mechanism and our results imply that a deficiency in apoptosis would have important consequence on skin cancer incidence. However, this have to be taken with caution mainly because the study has been conducted on fibroblasts and skin cancer occurs on keratinocytes and melanocytes. Further research would have to be performed to determine whether the apoptosis is also the sole UVB-induced cell death mechanism in keratinocyte. Moreover, PARP dependent metabolic response changes induce by UVB exposure need to be further investigated. Indeed, it appears to play an important role in response to genotoxic stress and might be implicated in UVB-induced carcinogenesis. Altogether, these data provide new insight on genotoxic response to lethal doses of UVB. This study shed some light on the interconnexion between DNA damage, metabolic response and cell death after UVB irradiation.

## Supplementary information

Supplementary Information.

## Data Availability

No datasets were generated or analysed during the current study.
