# Supplementary material for: Apoptosis, the only cell death pathway that can be measured in human diploid dermal fibroblasts following lethal UVB irradiation
Source: Sci Rep. 2020 Nov 3;10:18946. doi: 10.1038/s41598-020-75873-1 (PMC7609555; doi:10.1038/s41598-020-75873-1)
Supplement: Supplementary file 1 — Supplementary Information. [file 41598_2020_75873_MOESM1_ESM.docx]

**Supplementary material**

***Apoptosis, the only cell death pathway that can be measured in human diploid dermal fibroblasts following lethal UVB irradiation***

Anne-Sophie Gary^1,2^ and Patrick J. Rochette^1,2, 3,^*

1. Centre de Recherche du CHU de Québec – Université Laval, Axe Médecine Régénératrice, Hôpital du Saint-Sacrement, Québec, Qc, Canada

2. Centre de Recherche en Organogénèse Expérimentale de l'Université Laval/LOEX, Université Laval, Québec, Qc, Canada.

3. Département d’Ophtalmologie et ORL - chirurgie cervico-faciale, Université Laval, Québec, Qc, Canada.

*To whom correspondence should be addressed. Tel: (418) 682-7568; Fax: (418) 682-8000;
E-mail : Patrick-J.Rochette@crchudequebec.ulaval.ca

**Supplemental figures**

**
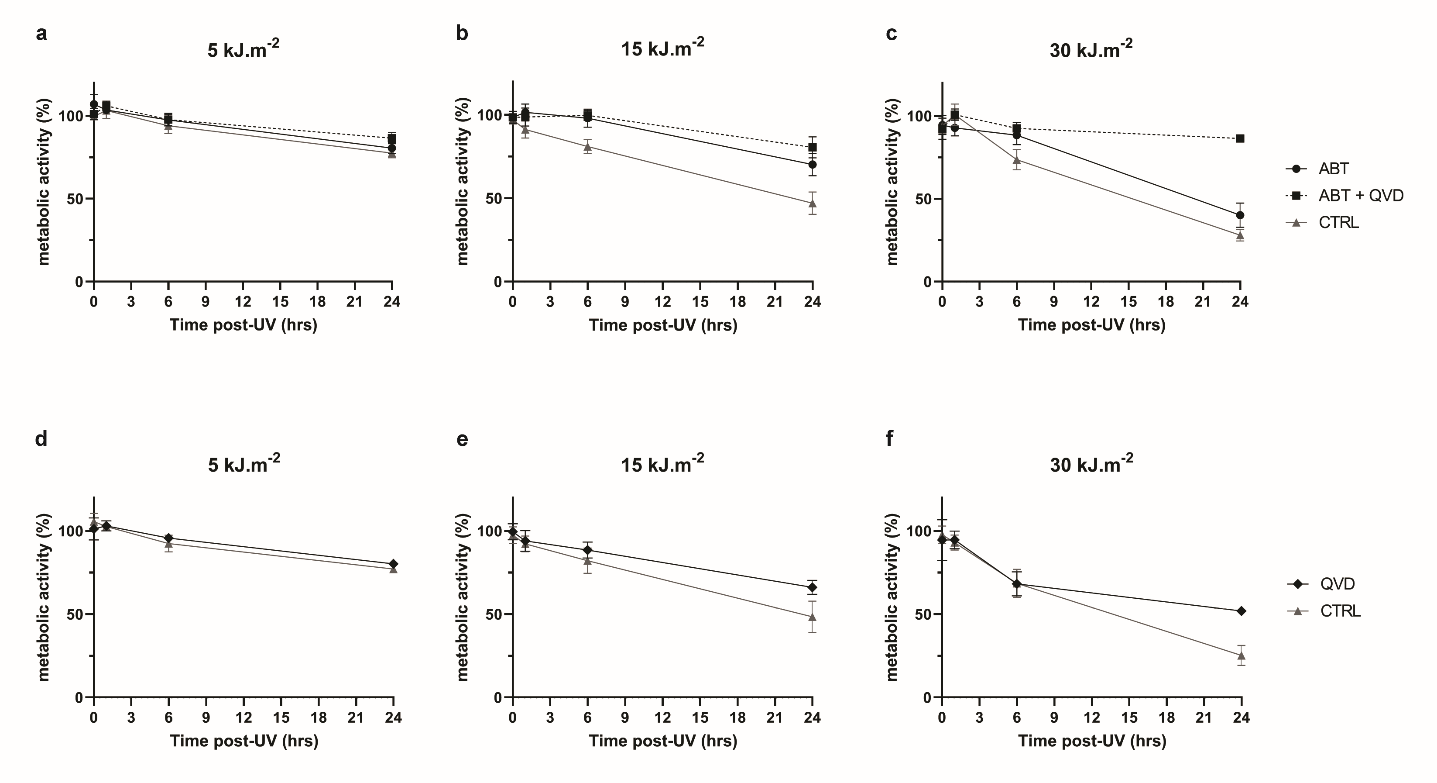
**

**Figure S1: UVB-dose dependent changes in metabolic activity**

Prior to UVB irradiation, NHDF were incubated 30 min with ABT888 (ABT, 20 uM), Q-VD-OPh (QVD, 20 uM) or the combination ABT888 and Q-VD-OPh (ABT + QVD). Cells were then irradiated in PBS using different lethal UVB doses: (**a**, **d**) 5 kJ/m^2^, (**b**, **e**) 15 kJ/m^2^ and (**c**, **f**) 30 kJ/m^2^. Cellular metabolic activity was assessed at different time points post UVB irradiation (0, 1, 3, 6, 24h) using MTS assay. Irradiated cells were normalised on unirradiated cells of the same condition. 5 kJ/m^2^ had little effect on cellular metabolism. At 15 and 30 kJ/m^2^, QVD significantly prevent UVB-induced cellular metabolic activity loss at 24h, ABT at 6 and 24h and the combination at 6 and 24h with an additive effect of QVD and ABT. N=4.

**Western Blot Full-length gels used for Figure 2**

**b**

**a**


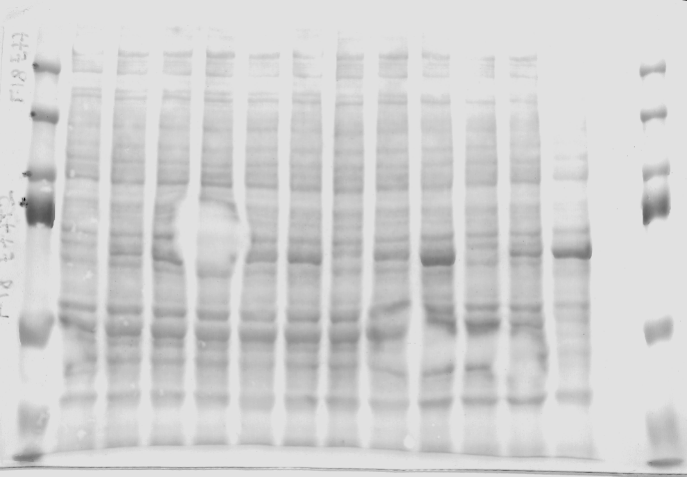

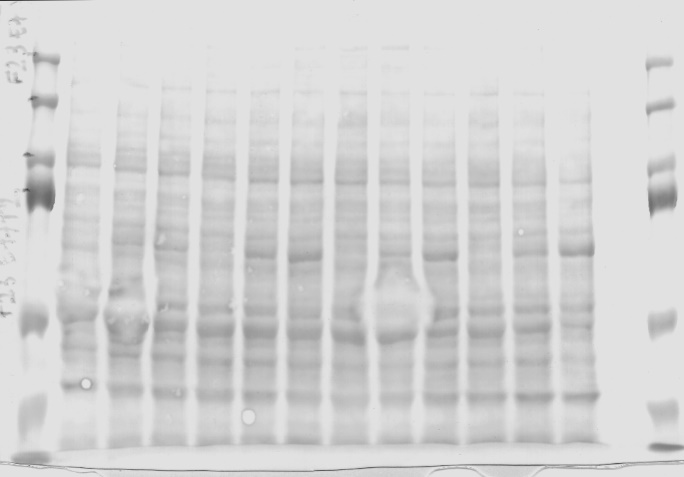


**d**

**c**


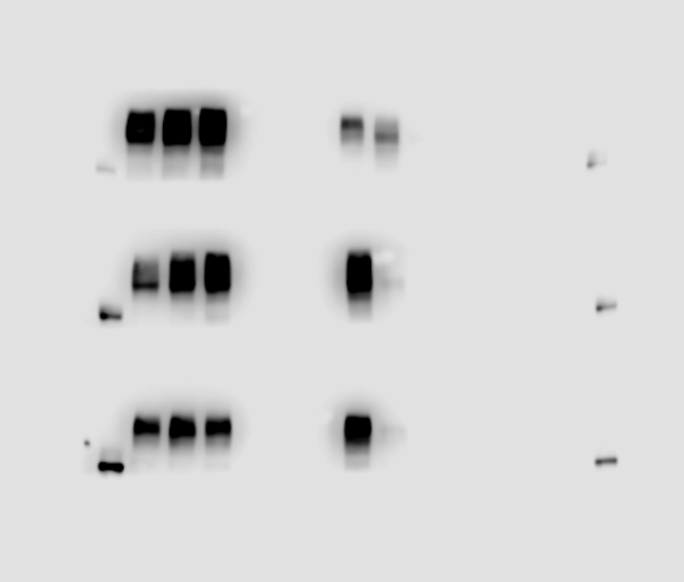

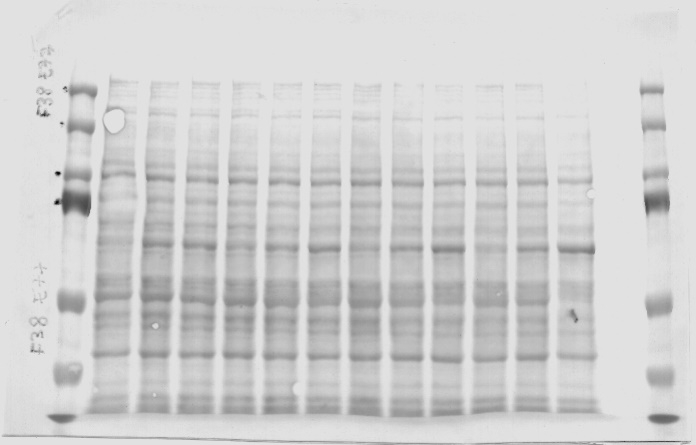


F18

F23

F38

**e**

**f**


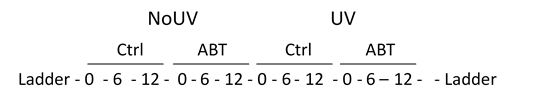

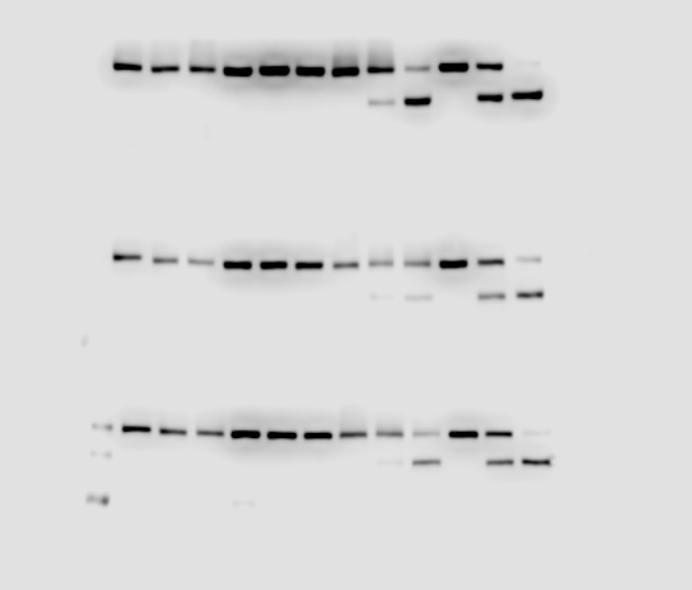


F23

F18

Loading control using Ponceau from F18 (**a**), F23 (**b**) and F38 (**c**). Membranes were cut at 150 kD using the molecular weight ladder. (**d**) The upper portion of the membranes were labelled using a PAR antibody. (**e**) The lower portion of the membranes were labelled using a PARP antibody. Samples order is show in (**f**). Data from F38 are presented in Figure 2.

F38

W**estern Blot Full-length gels used for Figure 6**

**a**

**b**


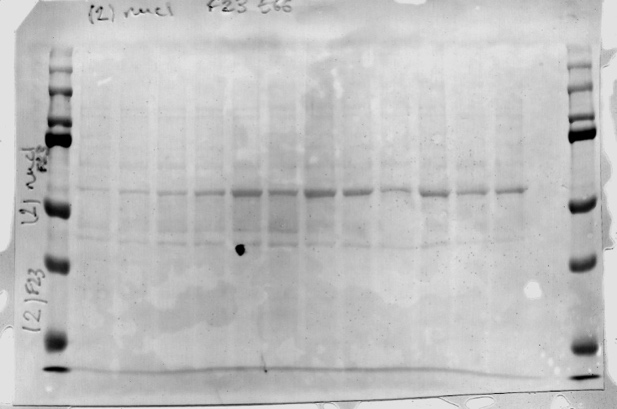

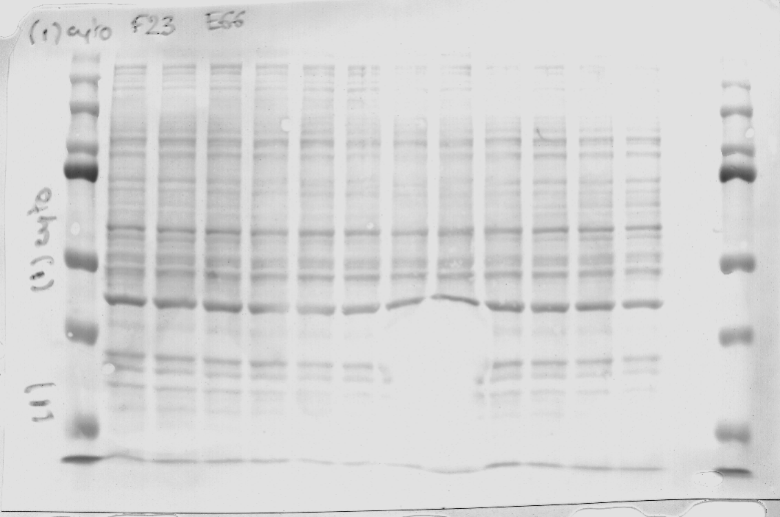


**c**

**d**


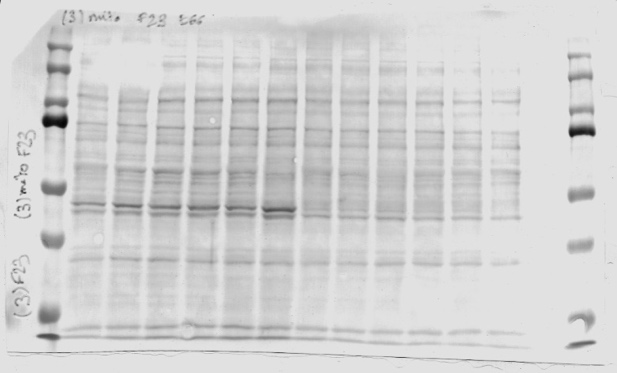


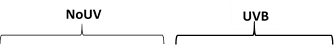


Ponceau staining has been used as loading control. (**a**) Ponceau from cytoplasmic fraction, (**b**) ponceau from nucleic fraction and (**c**) ponceau from mitochondria fraction. Samples order is show in (**d**).

**a**


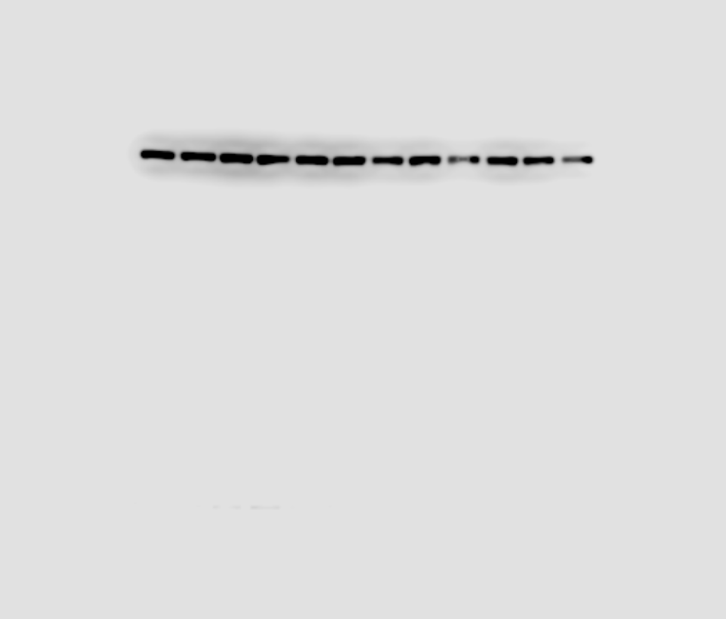

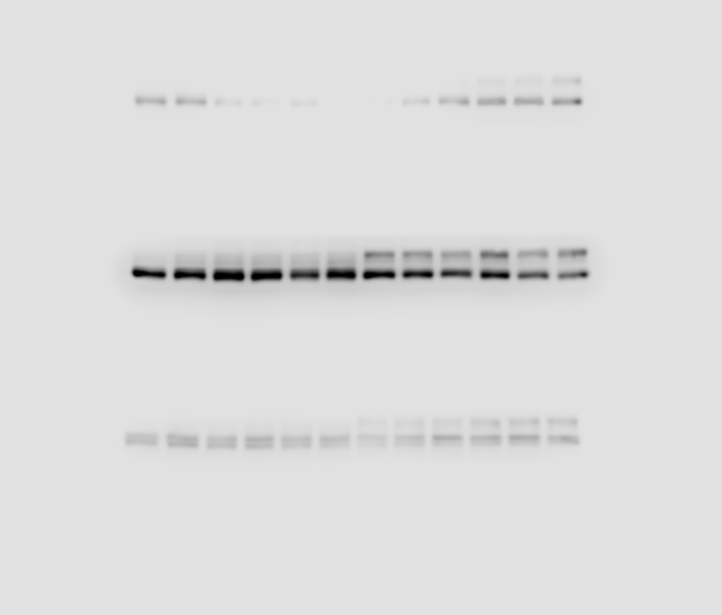


C

**b**

C

N

N

M

M

**d**

**c**


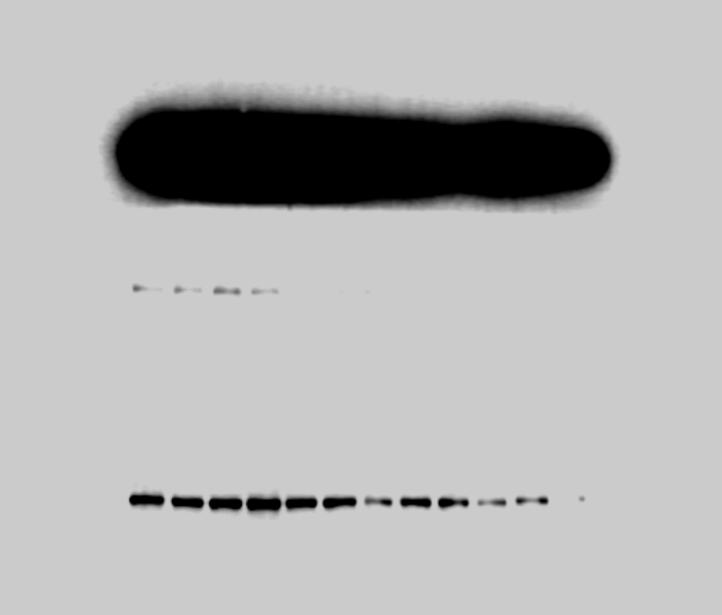

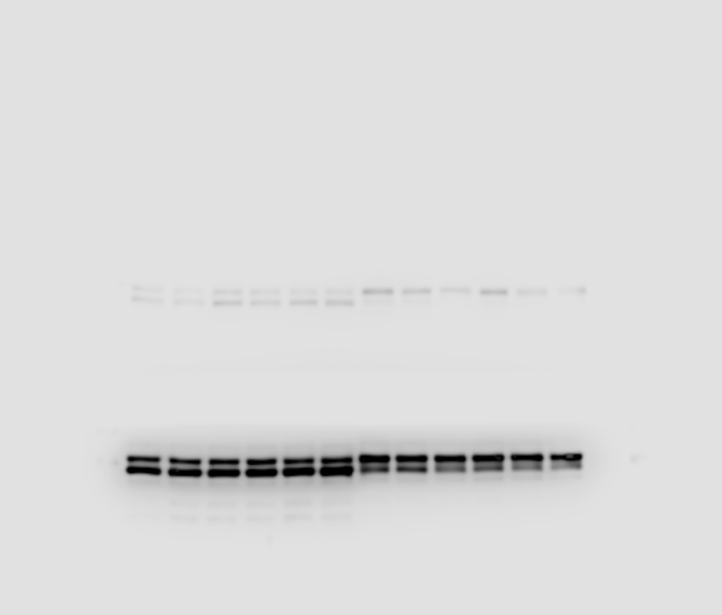


C

C

N

N

M

M

Membranes were cut between 37 and 75kD using molecular weight ladder. Controls of fractionation were measured by simultaneously scanning the membranes from the 3 fractions. Cytosolic (C), nuclear (N), and mitochondrial (M) membranes were place from top to bottom in the scanner respectively. Controls are (**a**) Lamin A/C for nucleus, (**b**) Tubulin for cytoplasm and (**c**) AIF for mitochondria fraction. (**d**) Over-exposition of the Tubulin showing the membranes from nuclear and mitochondria fraction.

**a**


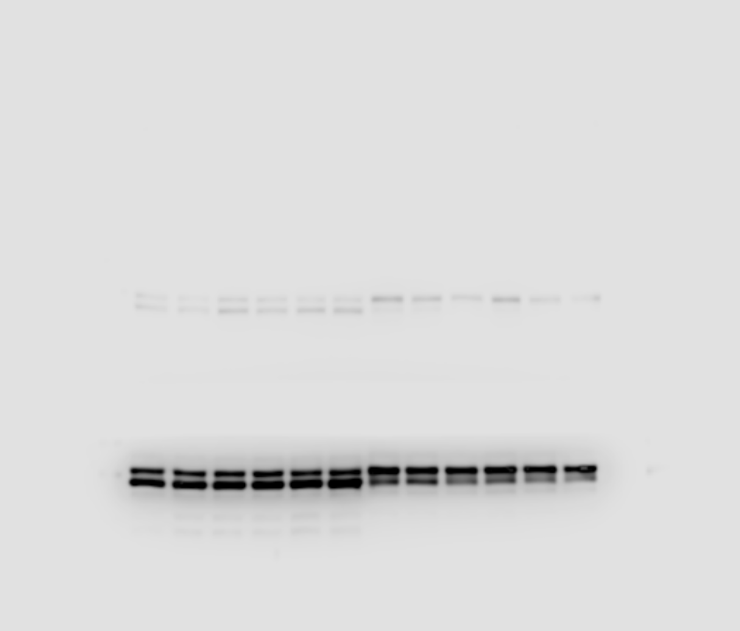


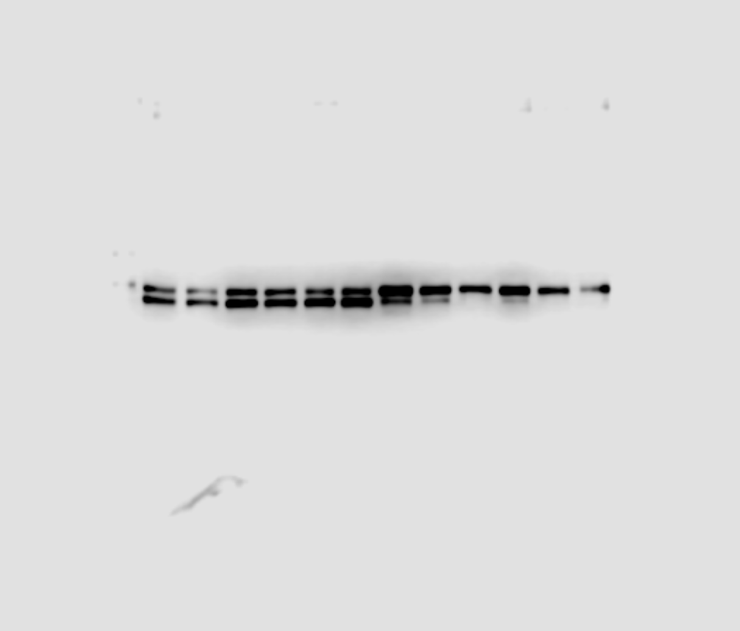


**b**

**c**


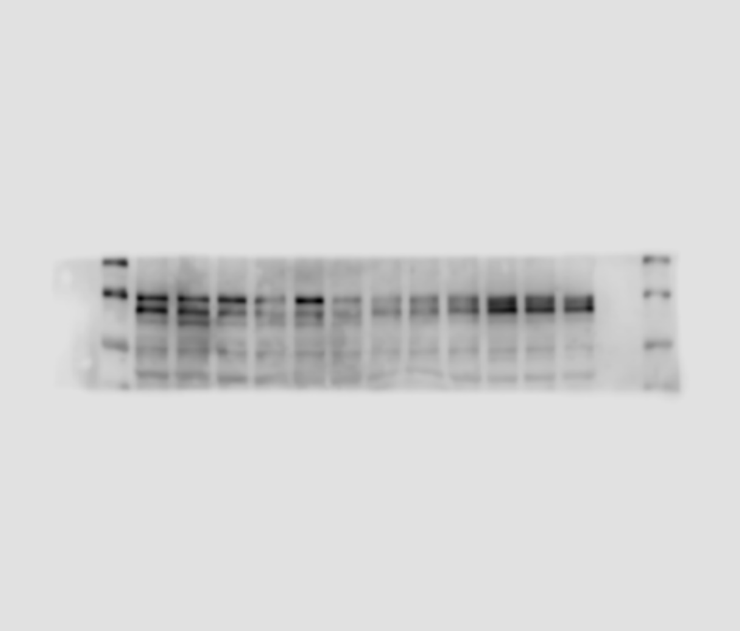


Mitochondrial control of fractionation was used to measure mitochondrial AIF (**a**). As nuclear and cytoplasmic AIF were weaker, nuclear and cytoplasmic fraction were scanned simultaneously showing nuclear AIF in (**b**). Membrane from cytoplasmic fraction was scanned alone to obtain AIF in this fraction (**c**).
